# Perceived changes in mothers’ outcomes following a pilot parenting program for couples with young children in Mwanza, Tanzania: A longitudinal qualitative study

**DOI:** 10.1371/journal.pgph.0006203

**Published:** 2026-10-06

**Authors:** Valencia J. Lambert, Alina Bhojani, Gabriel Sangarara, Julieth Joseph, Nathan T. Yang, Damas Joachim, Joshua Jeong

**Affiliations:** 1 Hubert Department of Global Health, Rollins School of Public Health, Emory University, Atlanta, Georgia, United States of America; 2 Tanzania Home Economics Organization, Mwanza, Tanzania; Baylor College of Medicine, UNITED STATES OF AMERICA

## Abstract

There is growing interest in engaging not only mothers but also fathers in parenting programs to improve nurturing care, psychosocial support, and early childhood development. However, few studies have explored how mothers perceive and benefit from participating in parenting interventions alongside their partners. This study explored mothers’ experiences during the pilot implementation of *Familia Bora*, a couples-based parenting program delivered in six communities in Mwanza, Tanzania (July-November 2024). In-depth interviews were conducted with mothers at baseline, midline, and endline using both random and purposive sampling. A total of 22 mothers participated in endline interviews, of whom 15 (68%) were purposively re-interviewed from the baseline sample to enable longitudinal qualitative analysis of perceived changes over time. Interviews explored mothers’ perceptions of changes in parenting practices, gender roles and attitudes, couples’ relationships, maternal mental health, and complementary feeding practices. Mothers commonly reported improvements across all domains. They described increased use of responsive caregiving and early learning activities, reduced harsh discipline, and improved nutrition knowledge and child feeding practices. Mothers also highlighted greater father involvement in childcare and more supportive couples’ relationship dynamics, which they perceived as reducing their caregiving burden and supporting their mental health. Overall, engaging fathers through couples-based parenting interventions may catalyze shifts within households that benefit mothers through improved caregiving practices, stronger partner relationships, and more nurturing family environments. These findings highlight the potential of father-inclusive parenting programs to support maternal, child, and family well-being.

## Introduction

Over the past several years, numerous parenting interventions have been implemented across diverse global settings. These interventions have improved early childhood development (ECD) outcomes by strengthening caregivers’ parenting knowledge, attitudes and practices [1,2]. Most parenting and early childhood development interventions have focused on inviting mothers [2]. While this approach has yielded important gains, it may also overlook opportunities to leverage broader family dynamics that shape caregiving practices and maternal well-being [3].

A growing body of research suggests that intentionally engaging fathers alongside mothers may enhance the reach and effectiveness of parenting interventions beyond mother-only programs [4]. Father-inclusive parenting interventions have been associated with improvements not only in paternal caregiving, but also in maternal caregiving, couple relationship quality, and ECD outcomes [2]. These effects may operate through multiple mechanisms. Increased father involvement in childcare and household tasks can alleviate the caregiving burden on mothers, thereby enhancing mothers’ capacity for caregiving and reducing maternal stress [5,6]. Additionally, studies suggest that behavioral changes are more likely to be sustained when both mothers and fathers participate in parenting interventions, as this promotes consistency in applying parenting strategies within the household and co-parenting roles and partner support [7,8].

Father engagement using gender-transformative strategies is particularly valuable in patriarchal contexts such as Tanzania, where fathers often hold primary authority in household decision-making [9] and exert considerable influence over maternal and child health, nutrition, and caregiving practices [2,10]. Involving fathers, in these settings, can help shift restrictive gender norms and promote more equitable sharing of caregiving responsibilities, household decision-making, and women’s empowerment, ultimately strengthening family environments that support both maternal well-being and child development [11]. Children tend to thrive in environments where caregivers collaborate in caregiving and maintain supportive, positive interactions with one another [12].

Building on this evidence, *Familia Bora* is a couples-based parenting program for caregivers, designed to strengthen parenting, partner relationships, and gender equity as family caregiving pathways to improving ECD outcomes. A pilot study was conducted to assess changes in both paternal and maternal outcomes. This manuscript focuses specifically on preliminary changes in maternal outcomes, as findings related to paternal outcomes are reported in a separate manuscript [13]. Using a longitudinal qualitative outcome evaluation design, we examine perceived changes in mothers’ parenting practices, gender roles and attitudes, couples’ relationships, maternal mental health, and nutrition knowledge and practices. This approach allowed for an exploration of the processes through which change may occur across these domains. By centering mothers’ voices, this manuscript explores how their perceptions and experiences evolved over the course of the *Familia Bora* intervention, including the ways in which fathers’ participation may have influenced their caregiving and family life. Overall, these findings contribute to a growing body of literature demonstrating how father-inclusive interventions can benefit mothers and ultimately the wider family unit.

## Methods

### Study setting

This study was conducted in six communities located in Ilemela District, within the Mwanza Region, one of the most densely populated regions in Tanzania. The six study communities were selected from Sangabuye Ward and Bugogwa Ward, both of which are categorized as rural wards, with average household sizes of 4.5 and 5.4 respectively [14].

Ilemela is a predominantly peri-urban district situated along the southern shoreline of Lake Victoria and is administratively divided into a mix of urban, semi-urban, and rural wards [15]. Livelihoods in these communities are shaped by proximity to Lake Victoria and surrounding peri-urban areas, with common income-generating activities including commercial fishing, small-scale agriculture, livestock keeping, and artisanal mining [16]. In Tanzania, women continue to bear a disproportionate share of household and domestic care responsibilities [17,18]. At the same time, restrictive gender norms influence expectations around caregiving, decision-making, and parental roles within families, although these norms are gradually shifting in some urban and peri-urban contexts [17,18].

### Program description

*Familia Bora* is a parenting intervention designed to support caregivers with young children by addressing interconnected domains of gender attitudes, parenting, couples’ relationships, and caregiver mental health [19]. The program, implemented from July to November 2024, included weekly group sessions lasting approximately 90 minutes. The pilot version of the program included 15 group sessions (**Table 1**), with two of the fifteen sessions conducted as father-only group sessions while the remaining 13 sessions are for couples.

**Table 1:** Structure of the *Familia Bora* sessions in the pilot version.

Session Number	Key Topic(s) Covered	Session Learning Objectives
1	Fatherhood (fathers only)	• Describe different common profiles of fathers • Reflect on personal definitions of “good fathers” • Reflect on how fathers’ upbringing shapes parenting beliefs and practices
2	Gender roles/attitudes in parenting	• Differentiate between sex and gender • Reflect on how gender norms shape caregiving and household responsibilities • Understand principles of gender equitable parenting
3	Early childhood development	• Understand the four developmental domains of early childhood development • Identify age-appropriate developmental milestones and skills
4	Responsive caregiving	• Define responsive caregiving • Practice responsive play and communication
5	Nutrition & responsive feeding	• Understand the key food groups and the importance of child dietary diversity • Reflect on household food consumption and purchasing patterns • Understand responsive feeding
6	Caregiver mental health (fathers only)	• Understand the impacts of stress on caregiver and child well-being • Identify common sources of stress for caregivers • Reflect on positive ways of managing stress
7	Power dynamics and partner communication	• Understand the differences between positive and negative forms of power in couples’ relationships • Distinguish between various communication styles • Practice positive partner communication
8	Early childhood development & responsive caregiving	• Understand the importance of play, communication, and responsive caregiving to improve early childhood development • Practice responsive play and communication
9	Intimate partner violence (IPV)	• Identify the various forms of intimate partner violence • Reflect on common sources of couples’ conflict and how to apply strategies to reduce conflict • Understand how couples’ conflict and intimate partner violence impacts women and children
10	Child discipline	• Differentiate between positive and harsh discipline • Apply positive discipline approaches and reduce use of harsh discipline
11	Play and learning materials	• Reflect on the various toys parents can make for their child • Practice making toys using locally available materials • Practice play and communication using different toys
12	Household budgeting	• Differentiate between spending on needs and wants • Reflect on the importance of saving and budgeting together as a couple to support the child and family well-being • Practice developing a basic household budget
13	Caregiver mental health	• Understand the impact of caregiver mental health and substance use on relationships with children • Reflect on healthy coping strategies to manage stress and reduce harmful behaviors
14	Fathers’ roles in nutrition & responsive feeding	• Apply responsive caregiving principles to child feeding • Describe fathers’ roles in promoting child health and nutrition
15	Closing session (recap of all sessions)	• Summarize key concepts across gender attitudes, parenting, couples’ relationships, and caregiver mental health • Reflect on behavior changes and plans for sustained practice

The *Familia Bora* curriculum was co-designed by the study team through an iterative process [19]. First, an initial review of evidence-based parenting and couples-focused interventions was conducted. This was followed by formative qualitative research with caregivers and community stakeholders in Mwanza to identify local needs and implementation considerations. In response to these findings, the study team developed new and original context-specific content and activities to address identified gaps, while also adapting some selected components from established fatherhood interventions shown to be effective in similar contexts [20,21]. Lastly, rapid piloting and refinement of session content and delivery approaches were conducted. Because this was a couples-focused program, intervention messages were intentionally tailored to resonate with both women and men and encourage shared learning on caregiving roles. Linkages between parenting practices, couples’ dynamics, gender norms, and caregiver mental health were emphasized through the program’s framing around caregiving for young children.

Two project coordinators from Tanzania Home Economics Organization (TAHEA), a local non-governmental organization, each holding a bachelor’s degree and with expertise in parenting and ECD, independently delivered group sessions in Swahili. Session times and locations were selected by participants within each community. Each session included delivery of content through facilitated discussions of key lessons, interactive activities, and skills-based practice. Sessions ended with a homework assignment intended to reinforce learning through application in the home environment.

Throughout the sessions, facilitators used a variety of materials that were locally developed by our study team, including flipcharts with illustrative images, toys and other play materials for children, and session-specific worksheets. To emphasize responsive caregiving content, facilitators also used a tablet to display videos and a play and communication guide with examples of activities for practice. Each household was also provided with a session-specific one-page printout which included a summary of the content covered and the homework assignment. Additional details about the program can be found elsewhere [22].

### Study design

We conducted a longitudinal qualitative study embedded within a broader pre-post mixed-methods evaluation designed to assess changes associated with the *Familia Bora* pilot on caregivers’ outcomes [13]. As part of the larger mixed-methods evaluation, a total of 62 households were enrolled, which included 10 couples (10 fathers and 10 mothers) in each of the six communities in Ilemela, with 12 fathers and mothers enrolled in one community due to high caregiver interest. Each of the six communities was selected in consultation with village chairpersons and reviewed by TAHEA. In each community, 10 households were randomly selected using simple random sampling from a list of eligible households compiled by the chairperson. Eligibility criteria required that primary caregivers were aged 18–65 years, primary caregivers had a child younger than 2 years of age, male caregivers were in a relationship with the child’s female caregiver, both caregivers resided with the index child during the past month, and written informed consent was provided. Results from the mixed-methods fathers’ impact evaluation are reported separately [13]. The current paper focuses on qualitative findings from mothers to explore their experiences and perceptions of the intervention. Quantitative findings were not collected from mothers as the evaluation was designed as a proof-of-concept study prioritizing fathers’ engagement, and available resources were therefore allocated to quantitative measurement among men only.

### Sampling and participants

Mothers were interviewed at baseline (July 2024), midline (September 2024), and endline (November-December 2024) using a combination of random and purposive sampling strategies. Combining these approaches enabled us to balance breadth and depth in the qualitative inquiry. Random sampling at baseline was used to capture a broad range of perspectives and experiences prior to program participation. Purposive sampling at midline and endline was subsequently used to ensure inclusion of mothers with varying levels of exposure to the intervention and to facilitate longitudinal exploration of perceived changes over time.

At baseline, 16 mothers (two to three from each community) were randomly selected for interview to capture a range of perspectives and experiences prior to the program. At midline, six mothers (one from each community) who had attended at least 50% of program sessions were purposively selected for interview. Mothers with greater exposure to the intervention were selected to inform program refinements and explore program experiences, adoption, and preliminary perceived changes. At endline, 22 mothers representing a range of program attendance levels (low, average and high) were interviewed to capture diverse experiences with the intervention. To facilitate a longitudinal qualitative analysis, 15 mothers interviewed at baseline were purposively followed up and re-interviewed at endline, while an additional seven mothers were added to ensure representation across final attendance profiles (Fig 1). The endline sample size of 22 mothers, including 15 with longitudinal data from both baseline and endline, provided sufficient power to address the study objectives. Interviews yielded rich accounts and recurring patterns across the major domains of interest, which supported the adequacy of the sample for analyses.

**Fig 1:**
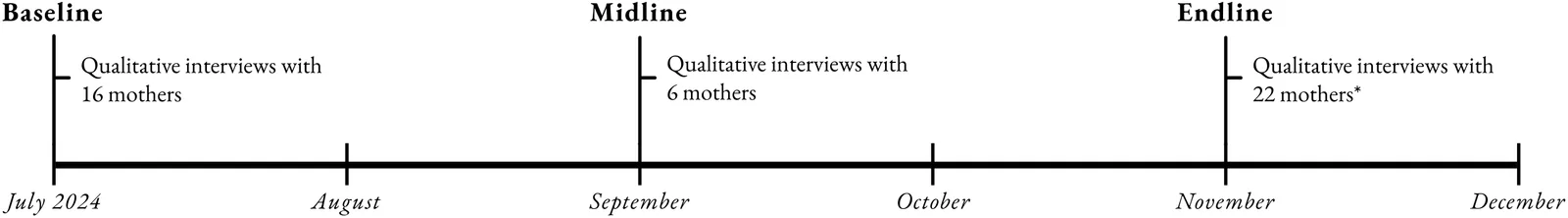
Overview of the qualitative sampling and data collected at baseline, midline and endline. *15 mothers from baseline re-interviewed at endline. 7 additional mothers were interviewed at endline.

### Data collection

A semi-structured interview guide was developed and adapted for each time point (baseline, midline, endline). Each topic guide was piloted prior to data collection with caregivers who were not included in the final qualitative sample. This process enabled refinement of the interview guide by incorporating more effective probes and revising questions to improve clarity. It also provided interviewers with an opportunity to familiarize themselves with the guide, thereby facilitating more natural and effective engagement with the participants. The in-depth interviews (IDIs) explored parents’ experiences participating in the program; perceived positive, negative or null changes across domains of caregiving, parent-child relationships, couples’ relationships, and caregiver well-being; and factors which helped or hindered behavior change. Example questions included: *(1) What did you learn about through your participation in the program? (2) How, if at all, has the program changed the way you care for your child? (3) How, if at all, has the program changed your relationship with your partner?* (4) *What specific aspect(s) of the program had the biggest impact in contributing to these changes? (5) Are there any lessons/actions you learned in the program but have not been able to do at home or see a difference in your life?*

IDIs were conducted in-person by two external Tanzanian research assistants based in Mwanza who were not involved in the implementation. Both were fluent in Swahili and English and had more than 5 years of experience in qualitative data collection in the Mwanza region. Each IDI took place in either private community settings or participants’ homes. They lasted approximately 60 minutes and were conducted in Swahili. Research assistants received comprehensive training which covered study protocols, ethical research practices, interviewing skills, and interview guide practice. Additionally, the research assistants were thoroughly oriented to the interview components. This included a review of the intervention materials and a comprehensive overview of all 15 sessions, including video recordings and photos showing the session structure and activities. This orientation ensured that the research assistants had a clear understanding of the program and were well positioned to interpret participants’ references to specific intervention components, particularly when discussing which aspects of the program had the greatest impact and participants’ overall experiences. All interviews were audio recorded and directly transcribed verbatim into English. Transcripts were reviewed by a bilingual member of the study team, fluent in English and Swahili, to improve clarity of the English translations, provide contextual clarification as needed, and ensure overall accuracy.

Additionally, demographic information was collected at baseline through a quantitative survey administered to fathers only. The survey captured basic characteristics of the father, mother, index child, and household/relationship. For the purposes of this analysis, only demographic information of the mothers interviewed as part of this qualitative evaluation is presented below, including characteristics related to the mother, index child, partner relationship, and household.

### Data analysis

#### Inductive-deductive approach.

Data were analyzed by a team of research analysts using a hybrid inductive-deductive approach. An initial codebook was developed collaboratively between analysts and refined based on initial coding of several transcripts and interview guide. It was further revised iteratively throughout the coding process to incorporate new codes that emerged from the data. Transcripts were analyzed using Atlas.ti Web by six analysts. To ensure coding consistency, all transcripts were double coded by two researchers. After line-by-line coding, the team conducted in-depth thematic content analysis, with four analysts preparing detailed summaries for each theme. Throughout the analysis process, the team held weekly meetings to synthesize findings across transcripts, highlight emerging themes, and jointly review results.

#### Framework method.

In parallel, a different team of four analysts applied the framework method [23] as part of the longitudinal analysis of the 15 mothers interviewed at both baseline and endline. These analysts reviewed transcripts line by line, summarizing findings within five broad domains in a matrix, where rows represented individual mothers and columns corresponded to the domains. These domains were informed by both the qualitative data and the intervention curriculum, and captured information on changes, or lack thereof, in parenting/ECD practices, nutrition knowledge and practices, gender attitudes, relationship dynamics, and mental health. To ensure accuracy and consistency in the level of detail captured in the matrix, two analysts involved in the qualitative coding reviewed all entries, cross-checking them against each transcript. Proposed revisions to the matrix were discussed and incorporated through weekly team meetings. Summarized findings for each caregiver (row) across domains (columns) were then compared between baseline and endline, and classified as indicating no, some, or substantial perceived change. “No change” reflected little or no reported difference between baseline and endline narratives within a domain. “Some change” reflected evidence of limited, emerging, or inconsistent changes in knowledge, attitudes, or practices, including partial uptake of program recommendations. “Substantial change” reflected clear, sustained, and frequently described changes in knowledge, attitudes, or behaviors that participants explicitly attributed to the intervention and that were supported by multiple examples within the interview. Classifications were based on analyst consensus following review of the full longitudinal case summaries and were discussed during team analysis meetings to ensure consistency.

#### Integration of analytical methods.

The framework analysis complemented the thematic analysis by enabling systematic within-participant comparisons of reported practices and knowledge before (baseline) and after the intervention (endline). While thematic code summaries captured group-level perceived changes, the framework analysis provided a structured assessment of the extent of change reported by individual participants across domains. Findings from the framework matrix were used iteratively during thematic analysis to identify deviant or negative cases, examine contextual factors associated with differing levels of perceived change, and assess how frequently particular patterns occurred across participants. For example, participants classified as demonstrating no, some, or substantial change were compared within and across themes to better understand mechanisms facilitating or constraining change. This integration strengthened the analysis by facilitating comparison of baseline and endline narratives to identify any inconsistencies or shifts in reporting, while providing a more structured understanding of the distribution and magnitude of perceived changes across participants.

### Reflexivity

The research team included Tanzanian and US-based members who worked closely to ensure contextual relevance. All members had experience working in the Mwanza region and/or in the implementation and evaluation of parenting interventions in similar settings. Approximately half of the team comprised Tanzanian researchers who were fluent in both Swahili and English and familiar with the local sociocultural context, which enabled nuanced interpretation of qualitative findings. To address potential bias, the team engaged in ongoing reflexive practices throughout the analytic process such as collaborative coding, joint development and refinement of the codebook, and regular meetings to discuss coding discrepancies.

### Ethics statement

This study received ethical approvals from the Institutional Review Board at Emory University and the National Health Research Ethics Committee at the National Institute of Medical Research, Tanzania. Written informed consent was obtained from all study participants. Additional information regarding the ethical, cultural, and scientific considerations specific to inclusivity in global research is included in the Supporting Information (S1 Checklist).

## Results

### Demographic characteristics

Of the 62 mothers enrolled in the *Familia Bora* intervention, 22 participated in IDIs across baseline, midline, and endline data collection waves. The median age of mothers was 28 years. Maternal education was relatively low, with only 14.3% of women having fully completed secondary school and none reporting education beyond secondary school. Most mothers were farmers (66.7%), followed by housewives (23.8%). The median age of the index children was 15 months, and a greater proportion were girls than boys (66.7% versus 33.3%, respectively). In addition to the enrolled index child, the majority of mothers (95.2%) had another child, with a median household size of 3 children. Most women were cohabitating with their partners but not formally married (90.5%), while 9.5% were legally or formally married. The median duration of cohabitation was 7 years (**Table 2**).

**Table 2:** Demographic characteristics of mothers included in this qualitative analysis (n = 21).

Sociodemographic characteristics	N (%) or median [IQR]
**Mother characteristics**	
Mother’s age (in years)	28 [26, 33]
Mother’s highest level of education	
No education	0 (0.0%)
Some primary school	2 (9.5%)
Primary school completed (or some secondary)	16 (76.2%)
Secondary school completed	3 (14.3%)
Mother’s occupation	
Farming	14 (66.7%)
Tailor	1 (4.8%)
Small business	2 (9.5%)
None (housewife)	5 (23.8%)
**Child characteristics**	
Child’s age (in months)	15 [7, 19]
Child’s gender	
Female	14 (66.7%)
Male	7 (33.3%)
Birth order	
First born	2 (9.5%)
Last born/Youngest in the household	17 (81.0%)
Other	2 (9.5%)
**Relationship/household characteristics**	
Marital status	
Legally or formally married	2 (9.5%)
Not legally or formally married but living with a partner in a consensual union	19 (90.5%)
Length of marriage/co-habitation (in years)	7 [3, 15]
Number of children in the household	3 [2, 6]

Note: Sociodemographic variables were collected at baseline from male partners only and are therefore reported by men.

Mothers reported changes across five broad domains aligned with the intervention curriculum: (1) Parenting practices, (2) Child nutrition (3) Gender roles and attitudes, (4) Couples’ relationships, and (5) Mental Health. The findings are presented in order of the most to least frequently discussed domain of change. Within parenting practices, mothers described increased play and learning materials within the household, improved stimulation practices, more responsive caregiving, and a shift away from non-violent discipline practices. In gender roles and attitudes, mothers described shifts in their perceptions of what constituted men’s and women’s respective responsibilities in the family. In couples’ relationships, mothers highlighted improved collaboration with their spouses in caregiving and household tasks, as well as shifts in gender role attitudes that supported more equitable sharing of responsibilities. Mothers also described improvements in communication and joint decision-making, and reductions in IPV, which collectively contributed to their overall satisfaction with their relationships. In mental health, mothers described how increased father involvement in caregiving and household tasks, combined with improvements in their quality of their relationships, positively influenced their overall well-being. They also described changes in their knowledge and use of positive coping strategies. Finally, in child nutrition, mothers described changes in knowledge and practices related to child feeding. Additional supporting quotations are presented in S1 Table.

### Parenting practices

Parenting practices were the most frequently discussed domain of change among mothers. Comparison of baseline, midline, and endline interviews suggested substantial shifts in parenting attitudes and practices over time, particularly regarding the importance of play and the use of harsh discipline. The program lessons prompted mothers to critically reexamine their past parenting practices, adjust their perspectives, and make meaningful behavioral changes to support their children’s early learning and development.

#### Theme 1: Mothers began prioritizing play and communication with their children and created homemade toys, which together enhanced the quality of their parent-child interactions.

Prior to the intervention during baseline interviews, mothers described their interactions with their children as largely limited to caregiving responsibilities such as bathing, feeding and supervising them. They did not prioritize or consider playing with their children an integral component of caregiving, with a few explicitly describing it as *“childish”*. Instead, their children either played alone or with their older siblings while mothers attended to work or other household responsibilities.

*“…. previously before I started attending the parenting sessions, I did not prioritize making time for playing with my child, the child could just be seated there crying and I could just be busy with my stuff leaving my child playing alone there until when I get done with my stuff. If not so, I would ask the older children to carry the baby or just continue with my work until when I am done then I take care of the baby...”* (Mother #2 at Endline, Village #4)

By endline, mothers described a notable shift in how they understood their role in supporting children’s development and recognized the importance of play in strengthening parent-child bonding and promoting early childhood development. Consequently, they reported intentionally setting aside time to play with their children. Several mothers also described making homemade toys, such as dolls and toy cars, from locally available materials, drawing on skills learned during the program and reinforced through homework assignments following the toymaking session. Notably, the ways in which mothers engaged with the toys varied. Some reported actively playing with their children using the homemade toys, while others simply provided them for independent play. Additionally, a few mothers described learning to incorporate safe and age-appropriate household materials in play, particularly when store-bought or home-made toys were unavailable. This enabled mothers to enrich their child’s play experiences even when financial and time constraints limited their ability to buy or make toys.

*“We learned so many things. Even how to make toys for children to play with and other play materials. For example, for boys, you can make a small car and show them how to play with it. If you do not make money, you can give them cans to play with, depending on their age.”* (Mother #11 at Endline, Village #3)

Beyond playing with toys, several mothers described integrating play-like interactions into their everyday routines, including talking with their children while doing household chores, singing to them, and storytelling. Communication emerged as a cross-cutting theme across these interactions, with mothers emphasizing that talking to their children using simple, age-appropriate language was itself a meaningful way to engage and stimulate their development.

*“All topics have contributed to these changes [in parenting practices]. For example the way I schedule my timetable, I should have time to know my child, for example, for the young baby who learn how to talk, a mother should not repeat the way he is pronouncing, you should pronounce well so that he knows the right word, slowly he will understand.”* (Mother #7 at Endline, Village #6)

Collectively, many mothers reported that this increased engagement in play and communication with their children, and the provision and use of toys, strengthened their parent-child bond and fostered greater enjoyment for both themselves and their children.

#### Theme 2: Mothers adopted responsive caregiving practices in their daily interactions with their children to support early learning and strengthen parent-child bonding.

Many mothers reported improved attentiveness to their children’s needs and cues, prompting them to adjust their parenting practices in response to the children’s preferences or signals of discomfort. Prior to the program, competing demands, particularly household and work-related responsibilities, limited their time for quality engagement with their children.

*“.... some people wish to spend time with their babies, talk and play, but income becomes the barrier, maybe [she] has no money at home, children are hungry, they cannot play with the child there is nothing inside, they will leave the baby with other children and go to find food. Every day they will concentrate on getting money, maybe she earns 5,000 per day and have 5 children, all the money is finished in a day, she has to find another one [money] for the next day.”* (Mother #7 at Endline, Village #6)

A few mothers also noted that, emotional cues such as crying were sometimes ignored or dismissed prior to the program. By endline, however, mothers came to understand crying not as disruptive behavior that needed to be managed but as a meaningful signal of varying needs such as a desire for closeness, comfort, or sleep. This reframing prompted mothers to become more attuned to their children’s cues and exploration initiatives such as attempts to crawl and talk. In response, they described providing toys to support exploration, talking to their children, guiding their actions, and playing alongside them.

*“We were taught to set aside time for the child and to care for what they need at the appropriate time. Sometimes a child may cry because they want to sleep, other mothers say, “This child is bothering me,” but the child has their needs, they need to sleep, so they may cry. Many parents do not know this. So, when a child cries, they see it as if the child is bothering them... But a child does not just cry, there is a reason, they could be unwell..”* (Mother #2 at Endline, Village #2)

Mothers frequently attributed the above changes to the videos and images used during the parenting group sessions. They noted that these program materials clearly illustrated how to interpret children’s cues and respond appropriately to their needs in various contexts.

*“For example, a child may cry, and you might force them to breastfeed even when they don’t want to. You find that the child wants to be carried on the back to sleep. There are other videos where a child doesn’t want to sleep, and you might force them. You carry them to sleep, but they don’t want to. The child wants to sit and play. This means the pictures and videos taught us that just because a child is crying doesn’t always mean they are hungry or want to nurse. Sometimes you just carry them and comfort them, and they fall asleep.”* (Mother #7 at Midline, Village #6)

#### Theme 3: Mothers increasingly adopted non-violent discipline practices, despite prevailing social norms that frame harsh discipline as acceptable.

Prior to the program, many mothers reported using harsh discipline, such as beating/hitting, shouting, scolding and using harsh language, because they believed it was effective in helping children learn from their mistakes and respect their parents. Compared with baseline interviews, many mothers reported at endline that the program lessons helped them recognize that their use of harsh discipline only instilled fear in their children.

*“I have learned many things, especially about children. It is possible to talk to a child and they understand. You can use force to talk to a child and still they will not understand you. I know that if you use force, a child will not respect you but will be fearing you...... It was easy for me to panic and be harsh to a child, but I have changed, I used to think if I am harsh a child will respect me, but the truth is they are scared of me. Now my children are doing things without being scared of me.”* (Mother #1 at Endline, Village #4)

Some also noted that they learned that their children’s misbehavior is often a reflection of their developmental stage, as young children lack understanding, are still exploring their environment through play, and may easily forget tasks they have been asked to do. This reflection allowed mothers to acknowledge how their past use of harsh discipline was inappropriate.

*“Initially, I was very strict in raising children. I would guide a child as if I were instructing an adult, but that is not the case because a child is too young and does not understand much. You need to be patient with them. For example, if a child did not understand, I would beat them. If they spilled water, I would beat them. But they were just playing. Through this project, I have changed a lot. When I recall what I used to do, it pains me, but I am happy because I have been transformed… I have realized that living with children is not about beating or scolding them, it’s about guiding them, and they understand.”* (Mother #9 at Endline, Village #5)

Changes in mothers’ perceptions of effective discipline techniques contributed to reductions in the use of harsh discipline including physical punishment (e.g., beating, hitting using sticks, slapping) and verbal punishment (e.g., scolding and using harsh language). Instead, mothers described learning and adopting more developmentally appropriate and non-violent strategies including guiding children calmly, patiently explaining negative behaviors and offering comfort when needed.

*“The way I used to discipline them by beating them, these days I just call them by words. But in the past, I was a very strict disciplinarian. Every mistake they made, I would beat them. Now, I just call them and say, “I don’t like it when you do this,” or sometimes I leave them and just watch them. But if they repeat the same mistake, I call them and say, “I don’t like you doing this.””* (Mother #6 at Endline, Village #1)

These changes were not limited to the index child (child enrolled in the program). A few mothers also reported using more positive discipline strategies with all their children and relying less on verbal or physical punishment.

None of the mothers interviewed explicitly reported that their discipline practices or views remained unchanged. However, some mothers acknowledged that prevailing social norms continue to frame harsh discipline as acceptable and effective in their communities, which may make it difficult for others to adopt alternative techniques promoted through the program.

*“The topic concerning punishing children, that issue is a major concern to many parents, most parents prefer punishing children with sticks rather than using polite or calm ways…the issue of avoiding punishing children severely or stopping beating them with a stick, that topic is very difficult to be implemented… they [parents] say that “without beating children they cannot behave well.”* (Mother #2 at Endline, Village #4)

### Child nutrition

Following parenting, the second most frequently discussed domain was child nutrition. Longitudinal comparisons suggested that improvements in nutrition knowledge and complementary feeding practices emerged progressively as mothers applied lessons learned through the program. Mothers described gaining knowledge about the importance of dietary diversity and learning to recognize and respond appropriately to their child’s cues of hunger, fullness and food preferences in ways that encouraged healthy eating habits.

#### Theme 1: Mothers reported improved nutrition knowledge and began prioritizing balanced diets for their children.

Most mothers described improvements in their nutrition knowledge, particularly regarding the importance of providing a balanced diet for their children. They reported learning about incorporating foods from diverse food groups such as fruits, groundnuts, and millets into their children’s diet. Beyond food selection, mothers also described learning about food preparation and feeding practices including avoiding overcooking vegetables, and adhering to recommended feeding practices such as exclusive breastfeeding for the first six months. In turn, they applied this knowledge to actively diversify their children’s diets to include proteins, fats, vegetables, and fruits, moving away from their repeated reliance on starch-heavy staple foods, such as ugali and rice, that dominated their diets before the program.

*“When it came to food, if the child was young, I would just give them porridge. Ugali and our usual dagaa, that’s how we lived... If I tried to change the meal, the best I could do was cook rice, full stop. Then this project came, and when it was introduced, our teachers started discussing things. [Now] I make nutritious porridge I mix starch, protein, millet, sesame, and soy in balanced portions.”* (Mother #8 at Endline, Village #6)

Notably, these dietary changes were not limited to the index child but benefited the entire household, including older siblings, as meals are typically shared as a family. Some mothers reported observing weight gain among their index children and perceived improvements in their older children’s energy levels and concentration in school, which they attributed to changes in household nutrition practices.

*“… the first change I noticed is that my child gained weight after learning about child nutrition. For instance, some people give their children the same porridge from grains every day, mixing the same things in the same way. For example, even rice and regular maize, they are essentially the same thing, but you end up loading the child with a lot of the same things. If you give them starch, it’s just starch. If it’s protein, after Madam [the facilitator] guided us, I followed up with her after the session. I asked for advice, and she helped me. Honestly, after that, when I took my child to the clinic, their weight increased.”* (Mother #7 at Midline, Village #6)

However, a few mothers acknowledged that financial constraints limited their ability to consistently provide a variety of foods to their children. Despite this, mothers described several strategies to navigate these financial barriers. Some reported redirecting the travel allowance provided by the program toward purchasing nutritious foods such as fruits, effectively offsetting some of the cost. Others noted that the lessons on budgeting equipped them with skills to allocate money efficiently. By writing down the household needs and assigning costs to each item, they came to realize that incorporating nutritious foods was feasible even with limited funds.

*“I was given a homework of preparing a family budget that indicates expenses on food items so that I could share it back to the following session. So I took rice and beans and watermelon, of which I spent like two thousand. On the session that followed I was asked to share my meal plan, and I stated it as I said earlier and I was congratulated that I included all important food categories in my meal plan. Yes, it was about food budget, as you know in our community some people might be admiring to have the very nutritious food, so when you balance the four food categories in a right proportion little by little at least it balances even with little budget [money].”* (Mother #11 at Midline, Village #3)

#### Theme 2: Mothers adopted responsive feeding practices to promote healthier eating habits in their younger children.

In addition to improvements in their children’s overall diet, some mothers reported changes in their feeding practices. They described adopting responsive feeding practices, such as singing and talking to their children during meals or eating together, to encourage their younger children to eat. This represented a stark shift from the techniques they previously used, either with the index child prior to the program or with older children, which had included force feeding or caning when children refused food. Mothers reported that unlearning these practices led to observable changes, with younger children beginning to accept and enjoy foods they had previously disliked, such as ugali. This reinforced the value of moving away from harsh discipline practices that had been normalized through their upbringing or social norms.

*“For instance, this younger child does not eat ugali, there was a lesson we were taught that you should observe what the child likes and not force them to eat something they do not like. So he does not eat ugali but eats other foods. I used to force him until I would hold him tightly to feed him against his will. Now that I have learned from this, I stopped doing that. I stopped and started showing him love. We used different methods to encourage him to eat, even what he does not like, by sitting together. When eating, do not isolate the child, sit with them, you might be eating while saying nice words to them. After I started doing that I could really see the changes to a child... Yes, you encourage him while feeding him, and when he eats, you praise him. I have used that method until today, my son eats ugali very well now, and he used to completely dislike eating ugali.”* (Mother #2 at Endline, Village #2)

### Gender roles and attitudes

Gender roles and attitudes was also among the top three most frequently discussed domains of change among mothers. Across interviews, mothers frequently contrasted their views before and after participation in the program, indicating shifts in attitudes toward fathers’ involvement in caregiving and household responsibilities They reported that the program lessons prompted them to rethink roles that they had unconsciously assumed due to social and gender norms, without ever having questioned them. These shared reflections prompted couples to work more collaboratively for the wellbeing of their families.

#### Theme 1: Mothers began to view caregiving as a shared responsibility between fathers and mothers.

Most mothers reported that *Familia Bora* led to meaningful shifts in gender roles within their families. Before the program, mothers consistently described clear divisions of roles within their households, with fathers mainly as the financial providers who were largely uninvolved in childcare or household activities, even when mothers desired this kind of support. One mother described:

*“The division of responsibilities is like when the mother is at home, doing household chores, while the father goes out to work and earn a living. That’s the division of responsibilities: the mother is doing housework, and the father is looking for ways to meet the family’s needs*.” (Mother #7 at Midline, Village #6)

A few mothers themselves reinforced these traditional divisions by expressing discomfort and even shame when initially introduced to the idea of fathers engaging in domestic responsibilities because they viewed such involvement as a sign that women were failing to fulfill their expected roles as mothers. In these accounts, resistance to shared caregiving was shaped by restrictive gender norms held by not only men but also women. For example, one mother reflected that program content on shared responsibilities was initially challenging for her to accept:

*“As I have said before that, there is a teacher who were washing clothes for his children, sometimes he washed even clothes for his wife, some people were saying that this is a best father, as he wash his children’s clothes, but on my side I was thinking that his wife does not deserve to be a wife, as she allows his wife to wash clothes... Yes, it was strange to me, but after I have been trained, I came to know that the teacher was right.”* (Mother #1 at Endline, Village #2)

However, after *Familia Bora*, nearly all mothers, including those who were initially skeptical of these ideas, described positive shifts in their gender attitudes. All women viewed fathers’ involvement as desirable and beneficial to family dynamics and easing disproportionate burden placed on mothers. At the same time, mothers situated their changing gender attitudes within a broader social context in which fathers’ involvement in caregiving continues to be stigmatized at the community level. While most mothers perceived growing acceptance within their own families, they described community norms as slower to evolve and occasionally resistant to change. For example, one mother described a situation where she overheard community members mocking her husband after seeing him carrying their child, explaining the father’s behavior as the result of being “bewitched” by his partner.

*“You know in the past men were very reluctant in helping their wives, even if you feel tired and ask for his help like of taking the child to clinic o hospital it was very difficult for them. When you see a man taking a child to clinic people might think of him in a negative way but currently as long as people have got more knowledge the issue of helping one another is becoming a common thing as people are more knowledgeable now. [Without being educated] they [the community] might think his woman has bewitched him.”* (Mother #3 at Endline, Village #1)

### Couples’ relationships

Another frequently discussed domain among the majority of mothers was improved couples’ relationships, which is closely intertwined with the previously described changes in gender roles and attitudes. Shifts in participants’ understanding of gender roles and the consequent renegotiation of household and caregiving responsibilities, were often reflected in and reinforced through changes in their relationship dynamics.

#### Theme 1: Increased father involvement in caregiving and household chores contributed to mothers feeling more supported, both practically and emotionally.

Prior to the program, mothers reported that when they asked their husbands to help with household chores or caregiving, fathers often rejected these requests by stating “*I cannot do women’s work”.* Several mothers added that even expressing a need for help could provoke conflict, leaving them feeling emotionally distant, ignored or disrespected. However, after the program, several mothers reported feeling *“confident”* making such requests, and these were met with a greater willingness from fathers. These changes in fathers’ involvement made mothers feel empowered and supported, both practically and emotionally.

*“In the past, if you asked a man to help with household chores, he would respond, “Ah, I cannot do that; I cannot do women’s work.” … But now, after being educated, they assist with chores, come home early, leave money for family needs, and play with their children… They have changed. In summary, you have helped us a lot.”* (Mother #11 at Endline, Village #3)

Specifically, several mothers reported feeling happy and valued as a result of their partners becoming more involved in caregiving and household tasks that previously fell on them entirely. This increase in father involvement contributed to a shift in their relationship dynamic, with mothers describing their relationships as filled with warmth, unity, mutual respect, and greater cooperation within the household.

*“Before the training, there was a lot of disrespect between us, and we were not close. But after the education, now we appear like a true couple because of the closeness we have built, and this has also increased the bond with the children. When the parents do not get along, the children also do not feel close to us.”* (Mother #7 at Midline, Village #5)

#### Theme 2: Mothers learned how to better navigate conflict and improve communication within their relationships.

At baseline, couples’ interactions were often strained, characterized by frequent quarrels, suspicion of marital affairs, and long periods of silence. Several mothers identified fathers as key contributors to miscommunication within their relationships, and as a result, mothers often avoided conversations with their partners altogether, either to prevent conflict or following an argument.

*“I have learnt how I am supposed to live with my husband, so I have changed a lot, I used to be angry most of the times when my husband wrongs me I could just stay angry even a week, not talking properly with my partner so I have been really educated and helped to change in that area also.”* (Mother #2 at Endline, Village #4)

However, through joint participation in the program, mothers and fathers learned how to navigate arguments more effectively, such as through communicating respectfully and talking through their problems, rather than resorting to harsh language or insulting words.

*“We were told to try to use good language or non-irritating or insulting words to our partners even if they have wronged us or dislike something about them, it is always a good idea to speak respectfully and kindly.”* (Mother #4 at Midline, Village #2)

Several mothers reported feeling more comfortable openly discussing various issues with their partners once fathers began listening to them and considering their perspectives. Mothers reported discussing a range of topics with their partners including finances, domestic/caregiving tasks, misunderstandings, and daily life. They reported that these improvements in partner communication brought them closer to one another, fostering mutual support and improving their collaboration in the home.

*“Now we have changed. Before the education, even if you wanted to talk to him, for example, “There is this problem at home,” he would not even listen to you. He would just leave without listening to anything you are saying. But now, even when you call him, he sits down, and you talk and discuss things together. Yes, if there is a problem, we discuss what to do. But before, he would not listen at all. If you told him, he would say, “You figure it out,” and he did not care.”* (Mother #3 at Midline, Village #1)

Few mothers felt that they did not experience substantial changes. Among them, one reported that improvements in communication were gradual, while another noted no changes in this domain. These mothers suggested that this was because the father was not physically present and not emotionally invested in their relationship, to apply the lessons learned in the program. *“There are still challenges. Most men here listen to other women outside more than their wives when it comes to relationships. Men here do not prioritize their wives. For example, my husband oversees fishing boats. His job is just to make phone calls and send money for household needs. But when it comes to the relationship, he has not changed...... Yes, without saying whether he will return or not. If you try to call him, he might not answer the phone or respond to messages.”* (Mother #4 at Endline, Village #2)

#### Theme 3: Mothers engaged in joint household decision-making with their spouses.

Mothers also described changes in household decision making over time. Prior to the program, many mothers described a lack of cooperation with their partners, with decisions often made individually by either the mother or father. For example, one mother described how her partner assumed the role of the “*head of the family*” and, as a result, made household decisions (e.g., selling of livestock, household expenses) on his own without consulting her and lied about it when she inquired. Additionally, before the program, many couples handled money independently, budgeted rarely, and lacked transparency about their income. Some mothers reported dissatisfaction with this lack of cooperation and noted that their attempts to improve collaboration with their partners, prior to the program, were often unsuccessful.

*“For example, in the past, when my husband had money, he would not involve me and hide his money… it really hurt me because we had money, but we were not cooperating. He would do it on his own, and I would just be surprised to see the result... [One day] I told him, “My husband, that is not how it should be. What you have and what I have should be combined so we can make a budget to clothe the children, buy food, and other things. But he did not understand.”* (Mother #9 at Endline, Village #5)

However, after the program, mothers reported making decisions jointly, particularly regarding household finances. They described openly discussing their income, sharing responsibility for expenses, and budgeting collaboratively. Importantly, these discussions were done respectfully; a stark contrast to before the program, when such conversations often led to arguments.

*“We would frequently argue, especially about money. If we saved money, it would get taken. My husband would take it [the money]. But now we have peace because of the lessons we have received. We now sit down together to plan our budget, what to save and what to spend…when he [the husband] attended the training, he learned a lot. I thank God because he has changed significantly. Now, when he comes home from work, he tells me the amount of money he has earned. We both put it on the table, and we have achieved a lot. Great things we did not have before through this project… within this short period, we have developed such mutual understanding that even when I am tempted to hide money now, my conscience troubles me.*” (Mother 9 at Endline, Village #5)

At the same time, a few mothers reported no changes in household decision-making, citing deeply rooted cultural norms in which household finances are primarily handled by fathers as a key barrier. One mother explained that despite her efforts to engage in more open discussions about finances, her husband remained unwilling to be financially transparent. His reluctance to be financially transparent, prompted her to withhold her own income as well. Collectively, these behaviors made it difficult for these couples to collaboratively manage household expenses.

***“****I think the issue of budgeting also is still a problem to me, we were taught together listening with our two eyes and our minds, but it depends on individual person perspective. For instance, my husband is working at the lake shore and I also work on my pet business. But you know we fail to disclose our income each one does not believe on his or her partner, even though I tried to open up to him ... So as a result, everyone is keeping his money and does not share anything about his or her income so that is how we live, we do not share our income, so this also is still a problem in my family between me and my husband.”* (Mother #2 at Endline, Village #4)

#### Theme 4: Mothers reported experiencing less physical, verbal and sexual violence from their spouses.

As highlighted above, several mothers reported that some discussions with their partners sometimes escalated into arguments, which were oftentimes filled with verbal insults. A few mothers also disclosed being hit by their partners during such conflicts. In response, these mothers described avoiding conversations or remaining silent, specifically because they felt unable to freely express themselves without any negative repercussions. After the program, mothers reported that their partners were less likely to resort to violence during arguments. Instead, mothers noted that fathers listened to their views, and disagreements were resolved more peacefully through joint discussion. As a result, these mothers described an increased sense of safety and emotional security within their relationships.

*“My husband was not a person who would listen to what a woman is saying, he was quick to beat, verbally abuse, mock. Even if you submit, he was violent but not now, he has changed. It is like we got married yesterday although we have children. When we have quarrels, we talk and correct each other. Back then when we fought the whole street would know. Now I have the ability to tell him if he is wrong and suggest what he can do right.”* (Mother #1 at Endline, Village #4)

In some cases, fathers’ heavy alcohol use was identified as a driver of violence, and reductions in alcohol-related aggression were noted in the broader context of reduced IPV victimization.

*“I am happy now since we got trained most men have really changed, even my husband was such a chronic drunkard and when drunk always was very furious when you talk to him. My husband was very cruel and very violent in our relationship, but nowadays he has changed a lot... Even if he continues drinking [alcohol], he has stopped issues of violence in our relationship. He can drink but when he comes back home, he comes with respect without any chaos and when I talk to him, he responds calmly and respectfully.... so nowadays even if he continues drinking alcohol, he does it responsibly and respectfully.”* (Mother #3 at Endline, Village #1)

The reduction in IPV extended to the sexual domain, suggesting the program shifted broader relational norms in which men’s sexual desires were prioritized over women’s. Prior to the program, a few mothers reported being coerced into sex or having their discomfort ignored. After couples jointly learned about consent and mutual respect, they experienced greater understanding from their partners, leading to mothers feeling more valued and secure.

*“There were times when I did not feel like engaging in sexual activity, and my husband would force me. He thought I was being stubborn, but I would feel unwell maybe my body was aching, I was tired, or I was sick. But you find that he used to insist, but through the training, he has fully understood. When I tell him I feel uncomfortable, he understands me... But now he is much better, he no longer exhibits that violence. He understands me completely. When I am tired, he understands”* (Mother #9 at Endline, Village #5)

### Mental health

Although mothers’ mental wellbeing was not explicitly probed in the interviews, discussions of stress and coping mechanisms emerged spontaneously in response to broader questions about the program’s impacts on caregivers’ lives. Mental health was therefore the least commonly reported outcome, with a smaller subsample of mothers describing related changes.

#### Theme 1: Several mothers reported improvements in their knowledge and application of positive coping strategies.

Among mothers who discussed about mental health, most frequently discussed coping strategies that they learned or adopted through the program. At baseline, only a few mothers were able to describe any positive coping strategies, which primarily included talking to their husbands or trusted family members. After the program, more mothers were able to describe a wider range of ways of managing stress including taking a break from situations to regain composure, practicing breathing exercises, and participating in religious activities as one mother described:

*“We were taught that if you are angry, leave and talk a walk, talk to your neighbor or a friend whom you trust or take a deep breath or go and cry to God. That’s what we were taught, and I loved it.”* (Mother #7 at Endline, Village #5)

Some mothers also described drawing on multiple coping mechanisms rather than rely on a single strategy, adjusting their approach depending on the situation and their needs at the time. At times, they used specific techniques, such as going for a walk, to reduce immediate stress, while in other situations they sought advice from trusted friends or family members for guidance.

*“We were taught various topics, and when you’re at home and facing stress, sometimes you just step out for a while, go for a walk, and by the time you return, your stress has reduced. Sometimes, if I’m feeling stressed or something is bothering me, I go to my mother-in-law and tell her, ‘Mama, this and this is bothering me. What can I do about it?’ The advice she gives helps, and there’s also my grandmother, with whom I talk and get advice.”* (Mother #6 at Endline, Village #1)

#### Theme 2: Strengthened partner and peer relationships improved several mothers’ mental health and psychosocial well-being.

A few mothers went further to describe how strengthened relationships with their partners and expanded peer support networks, including with other participants in the program, served as a source of psychosocial support that contributed to reduced stress, feelings of loneliness, and to greater overall well-being. Prior to the program, mothers frequently described limited or strained support from partners. Many noted how the lack of support and difficult relationship dynamics, including suspicion of marital infidelity, left them feeling isolated and was a significant source of emotional distress.

*“He [my husband] left home to do business and I was left with all responsibilities of supporting our family alone, I asked myself if I was married or just a single parent. When I told him that there were unfulfilled needs at home, he told me to fulfill them on my own. This got me really stressed… he takes money from home, sometimes without my consent and leaves nothing to support the family.”* (Mother #7 at Baseline, Village #1)

After the program, mothers described receiving greater emotional and practical support from fathers that helped them to feel less burdened and stressed. These improved partner relationships in turn helped them to feel more peace, happiness, and overall wellbeing in their lives.

*“Another thing I have experienced in this project is the issue of peace in my marriage, previous I was not living in peace in my family, but through this project I have peace now, I am living in good terms with my husband… We used to live in frequent chaos and quarrels, but now after we were taught in the project, we have learnt a lot and we live in peace as partners.”* (Mother #12 at Endline, Village #4)

Mothers also described how the program facilitated stronger peer relationships, which became important sources of emotional and practical support (i.e., advice) during challenging times. Although they didn’t often elaborate in detail the nature of these challenges, several mothers described that their parenting group had become “*like a family*” or as close friendships that provided them with a supportive and safe space to share personal challenges, seek advice, and relieve their stress.

At the same time, a few mothers noted that challenges within their relationships or daily lives had not fully disappeared. Nevertheless, they still emphasized that improved partner and peer supportive relationships helped reduce feelings of loneliness and emotional distress compared to before the program.

*“So sometimes I find myself like lonely when that happens as I do not get enough time to be with my husband and make some nice stories as partners...The challenge is still there but very little not as compared to some days back before we received some trainings.”* (Mother #4 at Midline, Village #1)

## Discussion

This longitudinal qualitative evaluation of the *Familia Bora* pilot program focused on mothers’ lived experiences identified improvements across five interrelated domains: parenting practices, gender roles and attitudes, couples’ relationships, mental health, and nutrition practices. Mothers consistently reported perceived changes not only in their individual behaviors but also in their relationships with partners, as well as improvements in their emotional well-being. Across domains, mothers frequently attributed these shifts to fathers’ joint participation in the program.

Engaging fathers in the program appeared to facilitate perceived relational and attitudinal changes around shared caregiving and parenting within the household that extended beyond mothers’ caregiving practices. Through guided discussions, fathers and mothers were encouraged to reflect on existing caregiving roles and gender norms within the household. These dialogues appeared to provide opportunities for many couples to reflect on, and in some cases, renegotiate traditional expectations around caregiving and household responsibilities, and consider more collaborative approaches to parenting. In addition, the program structure itself appeared to provide more opportunities for couples to interact, communicate, and practice new behaviors together. Through joint participation in play activities, group discussions, roles and take-home assignments, couples were encouraged to apply session lessons both during sessions and at home. Mothers commonly linked these shared experiences to perceived improvements in communication, couple’s relationships, and fathers’ engagement in caregiving and household responsibilities. Collectively, these aspects of the program were described as contributing to a more supportive psychosocial and family context, which mothers attributed to improvements across multiple outcomes.

Mothers reported perceived improvements in early learning and responsive caregiving behaviors. They reported greater engagement in a range of stimulation activities, such as making homemade toys, talking, singing and telling stories during daily routines, alongside more responsive interactions, including following their child’s lead and responding sensitively to their cues. Notably, mothers’ accounts of responsive caregiving were often more extensive and detailed than those reported by fathers [13]. Prior research in sub-Saharan Africa has consistently documented these gender divisions in caregiving, with mothers engaging more frequently in stimulation and caregiving activities than fathers [24]. These patterns reflect longstanding cultural norms that position mothers as primary caregivers who spend more time with young children [24,25]. In contrast, social expectations and economic pressures often position fathers as financial providers, limiting their time at home and reducing opportunities for interactions with their children ([26]. As a result, one possible explanation for these differences in caregiving involvement is that fathers typically spend less time with their young children, and efforts to increase their engagement in caregiving may require navigating additional barriers including restrictive gender norms and competing economic responsibilities [27].

In addition to stimulation practices, mothers also reported using fewer harsh disciplinary practices (e.g., hitting) and instead using more developmentally appropriate and non-violent approaches. In Tanzania, harsh discipline remains widespread and socially normalized [28], often reinforced through intergenerational beliefs about child rearing [29]. Mothers’ perspectives suggest that participation in the program facilitated reflection on these norms and introduced alternative strategies. Importantly, these changes were observed among mothers of children aged 0–2 years, underscoring the potential for early interventions to prevent harsh disciplinary practices, whereas much of the existing literature has focused on caregivers of older children [28].

Improvements in child nutrition knowledge and practices were also reported, particularly regarding dietary diversity and responsive feeding. Although mothers reported improved understanding that caregiving is a shared responsibility between mothers and fathers, and noted increased father involvement, responsive feeding practices were only reported by mothers. In contrast, fathers primarily focused on purchasing nutritious foods, with few examples of food preparation or direct feeding [13]. This difference may be influenced by social and gender norms, as well as economic pressures, which can discourage men and limit their participation in household tasks and caregiving [13,27].

Many mothers reported improvements in their relationship dynamics, including improved communication and reduced relational tension, such as fewer experiences of IPV. Mothers described these changes as contributing to a safer home environment in which they felt more valued, appreciated and empowered to express their opinions and concerns. Mothers perceived that improvements in relationship dynamics also contributed to their sense of emotional safety and overall relationship satisfaction.

While the majority of mothers described meaningful changes across the domains highlighted in this study, a minority reported limited changes in two areas: couples’ communication and financial decision making. A few mothers emphasized that changes in relationship dynamics are gradual, take time to materialize and are often dependent on fathers’ willingness to change. Mothers often attributed limited change in these areas to their partners’ unwillingness or inability to change behaviors, rather than to their own. This may reflect the prevailing gender norms in which joint decision-making may depend largely on men’s willingness to adopt inclusive practices, as women often concede to their partners in cases of disagreement [30]. These findings highlight how couples enter parenting programs at varying baseline relationship dynamics. Incorporating more individualized or targeted support within an intervention may help address persistent challenges by providing strategies specifically tailored to facilitate meaningful change.

## Conclusions

These findings suggest that there may be value in expanding the reach of parenting programs to include other caregivers beyond mothers. In addition to potentially improving maternal and paternal parenting knowledge, attitudes, and practices, couples-based parenting programs may provide opportunities for caregivers to reflect on restrictive gender norms and support more collaborative approaches to co-parenting and family caregiving relationships. Mothers in this study frequently perceived that increased father involvement and more collaborative couple relationships reduced caregiving burdens and contributed to a more supportive home environment in which their perspectives were more likely to be heard and valued. Evidence from studies in sexual and reproductive health, health promotion, violence prevention, and mental health further supports the potential effectiveness of engaging men to improve women’s outcomes [31], with several interventions demonstrating substantial benefits for women, including improved mental well-being [32,33]. This literature, together with the findings from the present study, suggests that father-inclusive approaches may hold similar promise in the context of parenting programs targeting caregivers of children aged 0–2 years.

This study has several limitations. First, the frequency of reports across domains may have been influenced by the prompts used in the interview guide. Unlike other domains, mental health had no explicit prompts to elicit responses on this topic. Therefore, the fact that it was the least frequently discussed domain may reflect this methodological gap rather than the program’s relative impact on this outcome. Additionally, mothers were invited to only one out of the two mental health sessions, limiting their exposure to this content compared to fathers. Future adaptations of the *Familia Bora* program could consider delivering all sessions, including the topics of mental health, to both mothers and fathers to ensure equal exposure across lessons. Second, this qualitative evaluation was conducted over a short follow-up period, approximately one month post intervention, which limits our ability to assess whether reported changes are sustained over time. Third, at midline low-attending mothers were not interviewed, which may have introduced a bias toward more favorable experiences. However, this subgroup was less than half of the total qualitative sample used in this analysis. Additionally, the findings above are presented with attention to the frequency with which themes were reported across the full sample size, which included mothers with low, medium and high attendance. This approach may have likely reduced the potential for overrepresentation of midline perspectives in the overall qualitative findings. Fourth, quantitative data were not collected from mothers as part of this pilot evaluation, which limited our ability to estimate the magnitude of reported changes at the group level. To strengthen the rigor of this qualitative evaluation, the study employed a longitudinal study design in which mothers’ narratives at baseline were compared with those at endline to strengthen credibility rather than relying solely on retrospective recall at endline. Future evaluations of the *Familia Bora* intervention should consider incorporating mixed-methods designs and particularly quantitative measures to more rigorously assess and triangulate findings about program impacts among mothers [34]. Fifth, social desirability bias may have influenced participants’ responses. Because mothers were discussing an intervention in which they and their partners had participated, some participants may have overstated positive changes or underreported ongoing challenges, particularly regarding sensitive topics such as parenting practices, gender roles, and couples’ relationships. To mitigate this risk, interviews were conducted by independent research assistants who were not involved in program implementation, and participants were encouraged to openly discuss both positive and negative experiences.

In conclusion, this pilot qualitative evaluation suggests that mothers perceived *Familia Bora* as contributing to changes in their caregiving practices, couples’ relationships, gender attitudes, nutrition practices, and psychosocial wellbeing. Findings highlight the potential value of multicomponent parenting interventions that address not only parenting and nurturing care, but also co-parenting, couples’ relationships, and gender norms. Mothers frequently described increased father involvement, improved relationship dynamics, and enhanced wellbeing as interconnected processes and perceived pathways through which they understood changes in their caregiving and family experiences. The findings support further investigation of father-inclusive parenting interventions and underscore the need for rigorous mixed-methods evaluations with longer follow-up periods to assess the extent, sustainability, and mechanisms of change among mothers and families in global contexts.

## Supporting information

S1 Table Additional quotes from mothers illustrating perceived changes of the program across key domains. (DOCX)

S1 Checklist Inclusivity in global research. (DOCX)
