# Supplementary material for: Perceived changes in mothers’ outcomes following a pilot parenting program for couples with young children in Mwanza, Tanzania: A longitudinal qualitative study
Source: PLOS Glob Public Health. 2026 Oct 6;6(10):e0006203. doi: 10.1371/journal.pgph.0006203 (PMC13641366; doi:10.1371/journal.pgph.0006203)
Supplement: Supplementary file 1 [file pgph.0006203.s001.docx]

**S1 Table. Additional quotes from mothers illustrating perceived changes of the program across key domains.**

| **Domain** | **Description** | **Illustrative quotations** |
| --- | --- | --- |
| Parenting practices | Learning from the program helped mothers recognize the importance of play, which improved the parent-child relationship. | *“Issues of parents playing with their children, we considered them to be like childish things, till when I knew the importance of that as it helps in improving relationship and closeness between parents and children.”* (Mother #4 at Midline, Village #2) |
|  | Mothers described making play materials and using them to actively play with their children. | *“I can also make some toy playing materials, so we are in very good terms with my child, even when I come back from my work I do make time to sit with my child and we have a time together to play.”* (Mother #3 at Endline, Village #1)  *“Regarding playing with children, it involves preparing toys, for example, cans. Dolls, balls made of cloth, you play with your child and guide them on how to play. You are playing together with your child.”* (Mother #4 at Endline, Village 2) |
|  | A mother described the importance of alternating toys to keep the child engaged. | *“Another thing is to prepare playing tools for the baby, for example toys, you give him things with impressed colors, he can also play alone and enjoy, not giving a cup to play every day that is not good.”* (Mother #7 at Endline, Village #6) |
|  | Mothers provided more learning materials and toys to help children achieve developmental milestones. | *“To motivate a child to walk, so my child delayed to crawl, when you put him somewhere he wouldn’t move, but motivating the child would make him move, you have to put playing tools away from him so that he become interested to move to them. After I received the training, I started making playing tools for him like dolls and coloring them will impress him to move, but now he is moving.”* (Mother #7 at Endline, Village #6)  *“I have changed and now I am very close to my baby, I take care of his needs, for instance now he is at a stage of practicing standing up so I made [asked a local carpenter to make it] a wooden car to support his standing practices and when he is tired I am able to notice and hence I do not force him and allow him to rest.”* (Mother #4 at Midline, Village #2) |
|  | Videos were relatable and helped parents learn and understand the concept of responsive caregiving. | *“The videos are very important as they teach and reflect what really happens in our communities.... There was a video that showed a mother trying to calm her child using various materials but the baby did not respond positively but when she tried to make the baby sleep the baby responded very well. In my opinion I think the videos are just okay the way they are. They are just good as well as they help us understand well what we are trained.”* (Mother #4 at Midline, Village #2)  *“There was a video of a parent that was showing his child a certain game of arranging glasses, another video was showing a child being directed by her parent to arrange a lid and a bowl. Yes, also paying attention to the child when he or she is trying to show you something and giving her or him whatever he or she desires and not forcing her or him to take or do something she or he does not want at that time, so generally listening and paying attention to the child.”* (Mother #4 at Midline, Village #4) |
|  | Mothers described learning about the importance of communicating with their child and practicing it even before their children could verbally respond. | *“One thing I have seen is whenever I communicate with my child, she can also respond back, even when I ask her to bring me something she responds by bringing it, so she can understand my language.”* (Mother #3 at Endline, Village #1)  *“One thing I have changed is speaking to my child. They are beginning to understand me even they cannot speak well yet, but they understand through gestures.”* (Mother #9 at Endline, Village #3) |
|  | Mothers’ reported changes from using harsh disciplinary practices to more positive discipline approaches (e.g., guiding the child, explaining negative behaviors, and offering comfort) | *“Another thing is the issue of how I respond to my child especially when there is something wrong the child has done, previously I used to punish my children very severely and angrily…. Yes, there is great change, previously when my child made a mistake I used to cane [hit using a stick] the baby but nowadays I might just call the baby and talk to him or her in a friendly manner and warn him or her in a friendly way with love”* (Mother #3 at Endline, Village #1)  *“Before I started attending the group sessions I did not believe in myself… I did not like beating my children but I would talk to them very harshly with insulting words… But through the training I have received, I have been educated and I have changed, nowadays I live well with my children, we make stories, we talk respectfully with my children.”* (Mother #2 at Endline, Village #4) |
|  | Mothers described reductions in harsh discipline for all children, not only the index child. | *“I care for my child better now and even for the older children, I have stopped hitting them, I explain to them what they should do and how they should care for the youngest child.”* (Mother #4 at Endline, Village #4) |
| Child nutrition | Mothers reported changes in their family’s consumption of nutrition foods, which in some cases resulted in visible improvements in their children’s health and development. | *“Before, my child was struggling in school. I realized that if a child is hungry, they can’t focus in class. When I started following up on their studies, I noticed that when they come home, they understand their lessons better. They say, "Mom, today we learned this and that in class." And I think, "Ah, so I was the one making their brains sluggish...... My younger child is also gaining weight. He used to weigh four-point-something kilos, and every time I take him to the hospital, he gains more.”* (Mother #8 at Endline, Village #6)  *“Honestly, I’ve changed in terms of nutrition… My children used to refuse to eat leafy vegetables. If I cooked them, they wouldn’t eat. They would say, "This isn’t food," and were used to eating fish and those simple foods. These days, they eat vegetables. We used to cook spinach, but we would overcook it until it’s soft. Now, we were taught how to cook spinach, how to cook beans, and now I see the difference. My children now eat vegetables, even my husband, who used to dislike vegetables.”* (Mother #6 at Endline, Village #1) |
|  | Mothers engaged in responsive feeding to better understand their child’s dietary needs. | *“As I said I am taking care of my child in the way did not do to other older children, previously… I could even breastfeed her [the older child] by force even if the child was not in need of.” (Mother #2 at Endline, Village #4)* |
|  | A mother described using the transport reimbursement provided by the program to overcome financial constraints of purchasing nutritious foods. | *“When I get the money, I cannot utilize it on my personal needs, I normally allocate it for my child’s needs, be it fruits, sugar, etc. and whatever remains I use it for personal needs.... even though we consider it as transport money, but I think it is mainly intended for improving the wellbeing of our babies on foods like fruits, etc.”* (Mother #11 at Midline, Village #3) |
| Gender roles and attitudes | Mothers felt supported when fathers began helping with childcare and household chores. | *“We do cooperate very well for instance in providing family needs… [my husband] usually helps me when he has time on activities like preparing meal while I also do some other issues like breastfeeding our baby. [Okay, so he was not doing all that in previous days?] Yes, I really feel so much good whenever he helps me.”* (Mother #4 at Midline, Village #2)  *“There are changes, he was lazy to baby sit a child, he would rather have me stop cooking and attend to a child, but now he has changed and knows that we should help each other. If I have work he takes a child and they go out so that I can work”* (Mother #5 at Endline, Village #6) |
| Couples’ relationships | Mothers described a shift in their confidence and ability to voice their opinions after fathers began respecting them more. | *“Before I didn’t have the freedom to tell him anything. In the past, we could even be coming from the farm, and I would ask him to help me carry a hoe, but he would refuse and give me his hoe, saying "Carry it yourself, and carry mine too." But now, even if I don’t ask him, he will tell me, "Give it to me, let me help you."* (Mother #6 at Endline, Village #1)  *“To us mothers, there are things that fathers see they are not respected if they do them to us. It helped us women to be confident and our men to listen to us.”* (Mother #1 at Endline, Village #4) |
|  | Mothers learned how to better navigate conflict and improve communication in their relationships. | *“I am living in good terms with my husband, but previously I had no peace that I have now with husband and in my marriage. We used to live in frequent chaos and quarrels, but now after we were taught in the project we have learnt a lot and we live in peace as partners.”* (Mother #1 at Endline, Village #4)  *“There is a difference, comparing before and now. I was different prior to the lessons, but right now, my partner and I have changed. He now listens to me and does what I tell him timely.”* (Mother #7 at Endline, Village #5)  *“My relationship with my husband has changed, as I have told you that previously we quarreled but nowadays we are ok after getting this training, because even when we have misunderstanding, we advise each other.”* (Mother #1 at Endline, Village #2) |
|  | Some mothers reported that fathers needed more time to change their communication style to improve the overall communication in their relationships. | *"We were taught that not every mistake needs severe punishment like beating or insulting your partner, it is always better to sit together and talk about the problem/ mistake in a friendly and calm manner rather than fighting, beating or talking harshly. Yes there is a change, though not that much big change but at least there is a change, you know change is always slow, it is not easy to speedily change a habit of a person overnight it is a gradual process and takes time and perseverance."* (Mother #2 at Endline, Village #4) |
|  | Mothers described increased involvement in household finances which included shared financial responsibilities, and budgeting for household expenses with their partners. | *“There were many topics, but budgeting stood out because we did not know much about it before. For example, I did not know how much I could spend in a day or how much to save. But we were taught that you can allocate a certain amount for weekly expenses and save another amount. Honestly, I found it very enlightening, and it has transformed me and my husband. We now practice budgeting.” (Mother #11 at Endline, Village 3)*  *“We share responsibilities. For example, I run a business, and my husband has his own business too, so when we share the duties, it works well. We help each other. If I am out of flour, I will take money and buy it, and if he notices there is no food, he will bring it. So, each one carries the family’s responsibilities.”* (Mother #7 at Midline, Village #5) |
| Mental health | Mothers described using coping strategies learned in the program to alleviate stress. | *“You may sometimes have stress; sometimes you even go out of the house, you just walk around, and when you sit there, things get better, and your mind becomes clearer.”* (Mother #7 at Midline, Village #6)  *“[Have you applied that at home?] Yes, we were told if you are stressed, you can even go to church and pray, or you can talk to a close friend you trust, and they will give you advice.* (Mother #3 at Midline, Village #1)  *“For example, when someone is stressed, they should leave the scene or call their relatives or friends to talk about their problem. Because when you talk about something or cry, you release what is weighing on your heart.”* (Mother #9 at Endline, Village #5) |
